# *Curvularia microspora* sp. nov. associated with leaf diseases of *Hippeastrum striatum* in China

**DOI:** 10.3897/mycokeys.29.21122

**Published:** 2018-01-18

**Authors:** Yin Liang, Shuang-Fei Ran, Jayarama Bhat, Kevin D. Hyde, Yong Wang, De-Gang Zhao

**Affiliations:** 1 Department of Plant Pathology, College of Agriculture, Guizhou University, Guiyang, Guizhou 550025, China; 2 Key Laboratory of Plant Resources Conservation and Germplasm Innovation in Mountainous Region, Ministry of Education, Guizhou University, Guiyang, 550025, P. R. China; 3 Guizhou Academy of Agricultural Sciences, Guiyang 550006, China; 4 No. 128/1–J, Azad Housing Society, Curca, Goa Velha, India; 5 Centre of Excellence in Fungal Research and School of Science, Mae Fah Luang University, Chiang Rai, 57100, Thailand

**Keywords:** China, hyphomycetes, identify, pathogen, taxonomy

## Abstract

An undescribed *Curvularia* sp. was isolated from the leaf spot disease of Barbados Lily (*Hippeastrum striatum* (Lam.) Moore). Phylogenetic analyses of combined ITS, 28S, *GPD1* and *TEF1* sequence data place nine strains of this species in the *trifolii*-clade, but they clustered together as an independent lineage with strong support. This species was morphologically compared with related species in the *trifolii*-clade. Based on differences in morphology and phylogeny, it is concluded that this species is a new taxon, introduced as *Curvularia microspora* **sp. nov.** Pathogenicity testing determined the new species to be pathogenic on *H. striatum*.

## Introduction

The genus *Curvularia* includes pathogens and saprobes of various plants, as well as opportunistic pathogens of humans and animals (Sivanesan 1987, Manamgoda et al. 2011, 2012, da Cunha et al. 2013, Hyde et al. 2014) and has been well-studied in recent years. Identification of *Curvularia* spp. was previously mainly based on morphological descriptions and comparisons, however, the use of molecular taxonomy has solved many problems of resolving species (Valente et al. 1999, Mendoza et al. 2001). A multi-gene phylogenetic tree, based on the internal transcribed spacers including the 5.8S nuclear ribosomal DNA gene (ITS), the 5’ end of the nuclear ribosomal large subunit (28S), fragments of the glycerol-3-phosphate dehydrogenase (*GPD1*) and translational elongation factor EF-1 alpha (*TEF1*) gene regions, was provided to identify fresh collections of *Curvularia* from various hosts and geographic locations worldwide (Manamgoda et al. 2015).

In this study, DNA sequences of ITS, 28S, *GPD1* and *TEF1* gene regions were used for phylogenetic analyses to identify a new *Curvularia* species. This was concluded based on the combined morphology and phylogeny. *Curvularia microspora* sp. nov., is introduced here, associated with leaf diseases of *Hippeastrum striatum*.

## Materials and methods

### Isolation and morphological studies

All diseased samples were collected from the Medical Plants Herb Garden, in Chongqing City, Nanchuan County, China. This garden is located in a region of subtropical humid monsoon climate and has conserved more than 3000 kinds of medicinal plants. In this study, all fungal strains were isolated by the single-spore technique in order to obtain pure cultures following the method of Chomnunti et al. (2014). Single spores were transferred to potato-dextrose agar (PDA) and incubated at room temperature (28 °C). After several weeks of incubation, the morphological characters were recorded following the methods of Manamgoda et al. (2011, 2012). Conidia and conidiophores were observed using a compound microscope (Nikon Eclipse E600 DIC microscope and a Nikon DS-U2 camera or a Nikon 80i compound microscope fitted with a Canon 450D digital camera). The holotype specimen was deposited in the Herbarium of the Department of Plant Pathology, Agricultural College, Guizhou University (HGUP). Ex-type cultures were also deposited in the culture collection at the Department of Plant Pathology, Agriculture College, Guizhou University, P.R. China (GUCC).

### DNA extraction and sequencing

Fungal cultures were grown on PDA until nearly covering the whole Petri-dish (90 mm) at 28 °C. Fresh fungal mycelia were scraped with sterilised scalpels. A BIOMIGA Fungus Genomic DNA Extraction Kit (GD2416) was used to extract fungal genome DNA. DNA Amplification was performed in a 25 μL reaction volume which contained 2.5 μL 10 × PCR buffer, 1 μL of each primer (10 μM), 1 μL template DNA and 0.25 μL Taq DNA polymerase (Promega, Madison, WI, USA). Primers ITS4 and ITS5 (White et al. 1990) were used to amplify the ITS region. The thermal cycling programme was: 3 min initial denaturation at 95 °C, followed by 30 cycles of 30 s denaturation at 94 °C, 30 s primers annealing at 52 °C, 1 min extension at 72 °C and a total 10 min extension at 72 °C. To amplify the *GPD1* gene, the primers gpd1 and gpd2 were used (Berbee et al. 1999). The amplification programme included an initial denaturation step at 96 °C for 2 min, followed by 35 PCR cycles with 1 min at 96 °C, 1 min at 52 °C and 45 s at 72 °C with a final 10 min extension at 72 °C. The *TEF1* and 28S regions were amplified using EF-526F/1567R and LR5/LROR primers respectively (Schoch et al. 2009). The 28S amplification programme included an initial denaturation step at 95 °C for 3 min followed by 30 cycles of 40 s denaturation at 94 °C, 50 s primer annealing at 52 °C, 1 min extension at 72 °C. The same PCR reaction was used to amplify *TEF1* with the only change being the annealing temperature at 54 °C.

### Phylogenetic analysis

DNA sequences from these isolates and reference sequences were downloaded from GenBank and analysed by maximum parsimony (MP) and maximum likelihood (ML) (Table 1). Sequences were optimised manually to allow maximum alignment and maximum sequence similarity, as detailed in Manamgoda et al. (2012). The alignment document of four phylogenetic markers has been submitted to TreeBase (https://treebase.org/; Accession number: 21970). A partition homogeneity test (PHT) was performed with 1000 replicates via PAUP v. 4.0b10 (Swofford 2003) to evaluate statistical congruence amongst sequence data of 28S, ITS, *GPD1* and *TEF1* gene regions. MP analyses were performed in PAUP v. 4.0b10 (Swofford 2003), using the heuristic search option with 1,000 random taxa addition and tree bisection and reconnection (TBR) as the branch swapping algorithm. Maxtrees were set to 10,000. The characters in the alignment document were ordered accordingly: 28S+ITS+*GPD1*+*TEF1*, with equal weight and gaps were treated as missing data. The Tree Length (TL), Consistency Indices (CI), Retention Indices (RI), Rescaled Consistency Indices (RC) and Homoplasy Index (HI) were calculated for each tree generated. Maximum likelihood (ML) trees of DNA sequences were obtained by a heuristic search using the TrN + I + G model, which was deduced as the best fit for the data by the likelihood ratio test using the MODELTEST wer3.7 and MrMTgui version 1.01 (Posada and Crandall 1998).

**Table 1.** GenBank accession numbers of isolates include in this study.

Species	Isolate	GenBank accesssion numbers and references							
		ITS		28S		*GPD1*		*TEF1*	
*Alternaria alternata*	EGS 34.0160	AF071346	Berbee et al. 1999	–	–	AF081400	Berbee et al. 1999	–	–
*Curvularia akaii*	CBS 318.86	HF934921	Amaradasa et al. 2014	–	–	HG779118	Madrid et al. 2014	–	–
*C. borreriae*	CBS 859.73	HE861848	da Cunha et al. 2013	–	–	HF565455	da Cunha et al. 2013	–	–
*C. borreriae*	MFLUCC 11-0442	KP400638	Manamgoda et al. 2015	–	–	KP419987	Manamgoda et al. 2015	–	–
*C. gladioli*	ICMP 6160	JX256426	Manamgoda et al. 2012	JX256393	Manamgoda et al. 2012	JX276438	Manamgoda et al. 2012	JX266595	Manamgoda et al. 2012
*C. gudauskasii*	DAOM 165085	AF071338	Berbee et al. 1999	–	–	AF081393	Berbee et al. 1999	–	–
*C. heteropogonis*	CBS 284.91	JN192379	Manamgoda et al. 2011	JN600990	Manamgoda et al. 2011	JN600969	Manamgoda et al. 2011	JN601013	Manamgoda et al. 2011
*C. ovariicola*	BRIP 15882	JN192384	Manamgoda et al. 2011	JN600992	Manamgoda et al. 2011	JN600971	Manamgoda et al. 2011	JN601020	Manamgoda et al. 2011
*C. pallescens*	CBS 156.35	KJ922380	Manamgoda et al. 2014	KM243269	Manamgoda et al. 2014	KM083606	Manamgoda et al. 2014	KM196570	Manamgoda et al. 2014
*C. ravenelii*	BRIP 13165	JN192386	Manamgoda et al. 2011	JN601001	Manamgoda et al. 2011	JN600978	Manamgoda et al. 2011	JN601024	Manamgoda et al. 2011
*C. trifolii*	AR5169	KP400656	Manamgoda et al. 2015	–	–	KP645345	Manamgoda et al. 2015	KP735694	Manamgoda et al. 2015
*C. trifolii*	ICMP 6149	JX256434	Manamgoda et al. 2012	JX256402	Manamgoda et al. 2012	JX276457	Manamgoda et al. 2012	JX266600	Manamgoda et al. 2012
*C. tripogonis*	BRIP 12375	JN192388	Manamgoda et al. 2011	JN601002	Manamgoda et al. 2011	JN600980	Manamgoda et al. 2011	JN601025	Manamgoda et al. 2011
*Curvularia* sp.	ICMP 10344	JX256444	Manamgoda et al. 2012	–	–	JX276455	Manamgoda et al. 2012	–	–
*Curvularia* sp.	ICMP 13910	JX256445	Manamgoda et al. 2012	–	–	JX276456	Manamgoda et al. 2012	–	–
*C. microspora* sp.nov	GUCC 6272	MF139088	This study	MF139106	This study	MF139097	This study	MF139115	This study
*C. microspora* sp. nov	GUCC 6273	MF139089	This study	MF139107	This study	MF139098	This study	MF139116	This study
*C. microspora* sp. nov	GUCC 6274	MF139090	This study	MF139108	This study	MF139099	This study	MF139117	This study
*C. microspora* sp. nov	GUCC 6275	MF139091	This study	MF139109	This study	MF139100	This study	MF139118	This study
*C. microspora* sp. nov	GUCC 6276	MF139092	This study	MF139110	This study	MF139101	This study	MF139119	This study
*C. microspora* sp. nov	GUCC 6277	MF139093	This study	MF139111	This study	MF139102	This study	MF139120	This study
*C. microspora* sp. nov	GUCC 6278	MF139094	This study	MF139112	This study	MF139103	This study	MF139121	This study
*C. microspora* sp. nov	GUCC 6279	MF139095	This study	MF139113	This study	MF139104	This study	MF139122	This study
*C. microspora* sp. nov	GUCC 6280	MF139096	This study	MF139114	This study	MF139105	This study	MF139123	This study

### Pathogenicity test

Pathogenicity of this species was determined by inoculating healthy leaves of *Hippeastrum striatum* and *Canna indica* L. with 5 mm diameter mycelial plugs, cut from the margins of 10-day-old actively growing cultures; the control was treated with sterile agar plugs. Both inoculated and control plants were kept in a moist chamber at 25 °C for 7 days and observed for disease symptom development. Infected leaves were collected and the fungus was re-isolated in PDA medium and compared against the original strains. Control plants were sprayed with sterilised distilled water.

## Results

### Phylogenetic analyses

**Figure 1.**
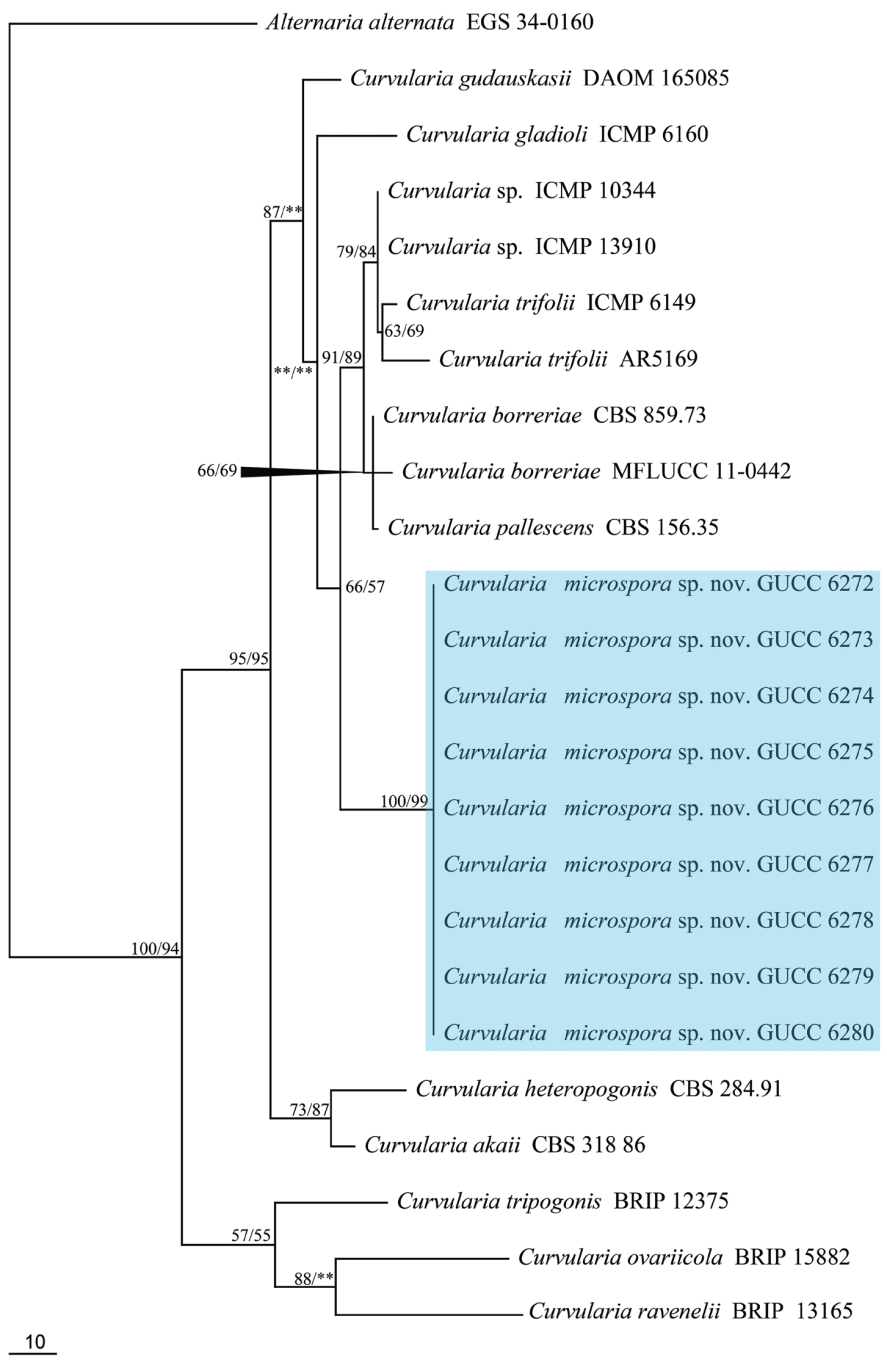
The only one parsimonious tree obtained from combined analyses set of ITS, LSU,β-tubulin and tef1 sequence data. MP values (>50 %) resulting from 1000 bootstrap replicates. The tree is rooted with *Alternaria alternata* (EGS 34-0160). The branch of our new *Curvularia* is shown in blue.

Nine isolates of *Curvularia* were sequenced from two plants in Chongqing Municipality, China (seven from *Hippeastrum striatum* and two from *Canna indica*). PCR products of approximately 900 bp (28S), 540 bp (ITS), 530 bp (*GPD1*) and 1200 bp (*TEF1*) were obtained. In the molecular phylogenetic analyses, the partition homogeneity test (P = 0.06) indicated that the individual partitions were not highly incongruent (Cunningham 1997) and thus 28S, ITS, *GPD1* and *TEF1* sequences were combined for sequence analyses. By alignment with a single gene region and then combination according to the order of 28S, ITS, *GPD1* and *TEF1*, only 2689 characters were obtained, viz. 28S: 1–848, ITS: 849–1330, *GPD1*: 1331–1771 *TEF1*: 1772–2689 with 104 parsimony-informative characters and 157 parsimony-uninformative characters. The analysis produced three equally parsimonious trees, one of which (TL = 366, CI = 0.81, RI = 0.82, RC = 0.66 and HI = 0.19) is shown in Figure 1 and the topologies of MP and ML analysis were congruent, thus only MP topology was shown. Phylogenetic analysis confirmed nine strains (GUCC 6272, GUCC 6273, GUCC 6274, GUCC 6275, GUCC 6276, GUCC 6277, GUCC 6278, GUCC 6279 and GUCC 6280) with the same DNA sequences in four phylogenetic markers grouped into an independent clade supported by high bootstrap values (MP: 100%; ML: 99%). These strains were placed in *trifolii*-clade with strong bootstrap support (MP: 95%; ML: 95%) and had a close relationship with *Curvularia gaudauskasii, C. gladioli*, *C. trifolii*, *C. borreriae* and *C. pallescens* with a high MP support (MP: 87%), but its ML bootstrap value was lower than 50%.

### Taxonomy

#### Curvularia microspora

Taxon classificationFungiPleosporalesPleosporaceae

Y. Liang, K.D. Hyde, J. Bhat & Yong Wang sp. nov.

822544

Figure 2

##### Diagnosis.

Characterised by producing four celled, smaller conidia (4.5–11.5 × 2–6 µm), usually curved at the third cell from the base.

##### Type.

China, Chongqing City, Nanchuan, from leaf spots of *Hippeastrum striatum*, 28 September 2016, Y. Liang, HGUP 6272, holotype, ex-type living culture GUCC 6272.

##### Description.

**Figure 2.**
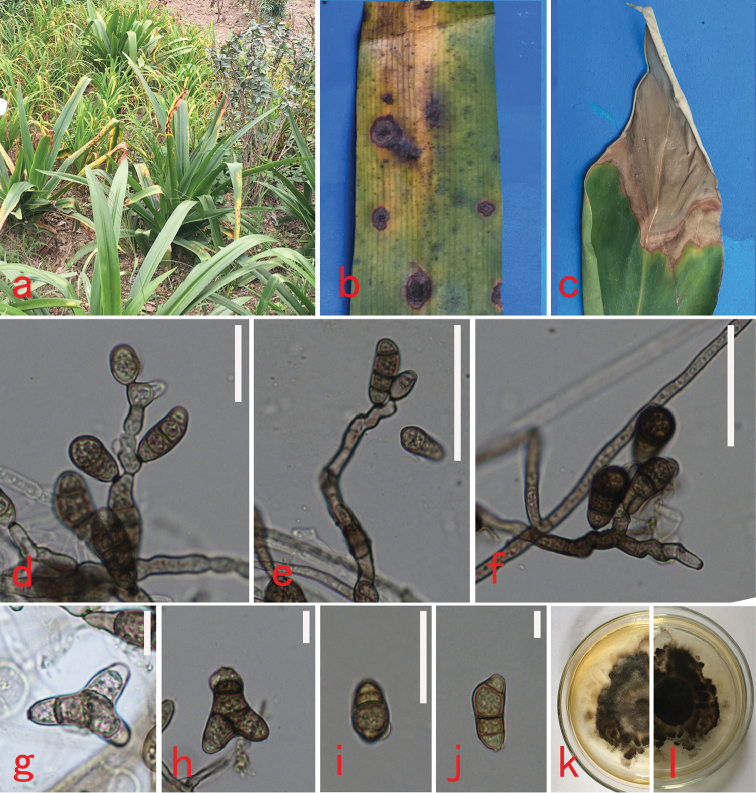
*Curvularia microspora* (HGUP 6272). **a–c** Leaf diseases symptoms on *Hippeastrum rutilum* and *Canna indica*. **d–f** Conidiophores, conidiogenous loci and conidia **g–j** Immature and mature conidia **k–l** Upper (**k**) and lower (**l**) surface of colony. Scar bars: **d, i** (10 μm), **e–f** = 20μm, **g–h, j** = (5 μm).

Symptoms on *Hippeastrum striatum*: Fructification mostly epiphyllous, disease spot 3–12 mm, subspherical to oblong ovate, brown to dark brown, effuse (Figure 2a, b). Symptoms on *Canna indica*: Fructification of the fungus was mostly epiphyllous, the large blighted, irregular spots near leaf apex to the whole leaves, greyish-brown (Figure 2c).

Colonies on PDA, vegetative hyphae septate, branched, subhyaline to brown, smooth to asperulate, 1.5–3 µm, anastomosing. Sexual morph: Undetermined. Asexual morph: Hyphomycetous. Conidiophores 10.5–77.5 × 1–3.5 µm (av. = 22.2 × 2.1 µm, n = 30), arising singly, simple or branched, flexuous, septate, geniculate at spore bearing part, pale brown, dark brown, paler towards apex. Percurrent proliferation only observed occasionally. Conidiogenous loci somewhat thickened and darkened, spores up to 0.8–1 µm diam, smooth. Mature conidia always four celled, 4.5–11.5 × 2–6 µm (av. = 8.2 × 3.8 µm, n = 50), smooth-walled, usually curved at the third cell from the base, sometimes straight, navicular, bifurcate, obpyriform, tapering towards rounded ends, pale brown to dark reddish brown. Hilum usually conspicuous or sometimes slightly protuberant.

##### Habitat and distribution.

Isolated from leaf diseases of *H. striatum* and *Canna indica* in China

##### Etymology.

*microspora*, referring to this species producing obviously smaller conidia.

##### Other material examined.

China, Chongqing City, Nanchuan, from leaf diseases of *H. striatum*, 28 September 2016, Y. Liang (HGUP 6273), living culture GUCC 6273; China, Chongqing City, Nanchuan, from leaf diseases of *H. striatum*, 28 September 2016, Y. Liang (HGUP 6274), living culture GUCC 6274; China, Chongqing City, Nanchuan, from leaf diseases of *H. striatum*, 28 September 2016, Y. Liang (HGUP 6275), living culture GUCC 6275; China, Chongqing City, Nanchuan, from leaf diseases of *H. striatum*, 28 September 2016, Y. Liang (HGUP 6276), living culture GUCC 6276; China, Chongqing City, Nanchuan, from leaf diseases of *H. striatum*, 28 September 2016, Y. Liang (HGUP 6277), living culture GUCC 6277; China, Chongqing City, Nanchuan, from leaf diseases of *H. striatum*, 28 September 2016, Y. Liang (HGUP 6278), living culture GUCC 6278; China, Chongqing City, Nanchuan, from leaf diseases of *Canna indica*, 28 September 2016, Y. Liang (HGUP 6279), living culture GUCC 6279; China, Chongqing City, Nanchuan, from leaf diseases of *C. indica*, 28 September 2016, Y. Liang (HGUP 6280), living culture GUCC 6280.

### Pathogenicity test

**Figure 3.**
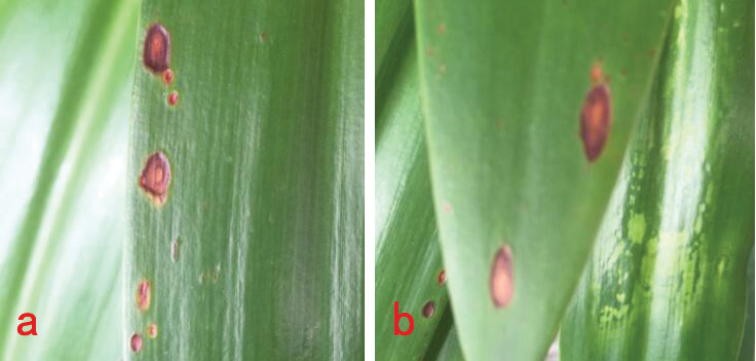
*Curvularia microspora* inoculated to *Hippeastrum rutilum* (7 days). **a** the first time for inoculation **b** the second time for inoculation.

Test plants (*Hippeastrum striatum*) were inoculated with 5 mm diam mycelial plugs of *Curvularia microspora* with two replicates of each plants and the inoculation experiment was repeated two times (with different sporulation generations). *Hippeastrum striatum* leaves both exhibited brown to dark brown necrotic spots (Figure 3a, b) after 7 days, which were very similar to those of natural infection (Figure 2a, b). The DNA sequencing result (ITS region), after re-isolation, identified this as *C. microspora*. The successful re-isolation of *C. microspora* from the inoculated leaves of *H, striatum* established a credible proof of pathogenicity. All test plants were covered with polyethylene bags for 7 days. However, on *Canna indica*, disease symptoms did not appear again.

## Discussion

The nine strains of *Curvularia* had typical characters of the genus., viz. the production of sympodial conidiophores with tretic, terminal and intercalary conidiogenous cells and elongate, transversely septate conidia with a dark basal scar (Boedijn 1933). Phylogenetic analyses compared the DNA sequence from four phylogenetic markers with related species in the *trifolii*-clade: *Curvularia akaii*, *C. borreriae*, *C. gladioli*, *C. gaudauskasii*, *C. heteropogonis*, *C. pallescens* and *C. trifolii* (Figure 1, Manamgoda et al. 2012, 2015, Madrid et al. 2014, Jeong et al. 2015, Su et al. 2015). These taxa are morphologically similar in producing a strongly protruding hilum (Madrid et al. 2014). However, the present taxon had bifurcate conidia, which differentiates it from all other species in the *trifolii*-clade. *Curvularia microspora* also has smaller conidia than the related species. A synopsis of the characters in the *trifolii*-clade is given in Table 2. The phylogenetic analyses (MP and ML) also confirmed these isolates belong to a new taxon with strong bootstrap support (Figure 1).

**Table 2.** Morphological comparison and pathogenecity of *Curvularia microspora* and related species in *trifolii*-clade.

Species name	Taxonomic references	Conidia		Conidiophores	Pathogenecity	Pathogenic reports
		Shape	Size range			
*Curvularia microspora*	This study	curved at the third cell from the base, sometimes straight, navicular, bifurcate, obpyriform, tapering towards rounded ends	4.5–11.5 × 2.0–6.0 µm	10.5–77.5 × 1.0–3.5 µm	Yes	This study
*Curvularia akaii*	Tsuda and Ueyama (1985)		24–34 × 8.7–13.8 µm		Yes	Zhang (2004)
*Curvularia borreriae*	Ellis (1971)		20–32 × 8–15 µm		No	
*Curvularia gladioli*	Boerema and Hamers (1989)		17.5–37.5 × 6.5–17.5 µm		Yes	Horita (1995); Torres et al. (2013, 2015)
*Curvularia gudauskasii*	Morgan-Jones and Karr Jr (1976)		27–29 × 15–19 µm	62–98 × 5–6 µm	Yes	Chinea (2005); Ratón et al. (2012)
*Curvularia heteropogonis*	Alcorn (1990)		27–44 × 11–19 µm	115–620 × 4–6 µm	Yes	Alcorn (1990)
*Curvularia pallescens*	Ellis (1971)		17–32 × 7–12 µm		Yes	Berg et al. (1995); Dadwal and Verma (2009); Mabadeje (1969); Rajalakshmy (1976)
*Curvularia trifolii*	Groves and Skolko (1945)		20–34 × 8–14 µm		Yes	Falloon (1976); Khadka (2016); Sarwar and Srinath (1965); Sung et al. (2016); Zamorski (1983);

*Curvularia* species can cause severe or opportunistic diseases of different plant taxa and are often a threat to agricultural production by reducing yield and quality. In the *trifolii*-clade, all species except for *C. borreriae*, have been reported as causing plant disease. This is especially true of *C. trifolii* and *C. pallescens*, which cause serious diseases of *Agrostis stolonifera* and *Gloriosa superba* respectively (Table 2). Koch’s postulates were performed to show that *C. microspora* causes leaf spot disease of *Hippeastrum striatum* (Figure 3), but on *Canna indica* might only be saprobic or endophytic. *Hippeastrum striatum* as an economic ornamental plant is grown in some areas of China, thus there is a need to continue investigation on the biology of this species in order to determine whether it can cause serious disease outbreaks.

## Supplementary Material

XML Treatment for Curvularia microspora
